# The Role of Microbial Inoculants on Plant Protection, Growth Stimulation, and Crop Productivity of the Olive Tree (*Olea europea* L.)

**DOI:** 10.3390/plants9060743

**Published:** 2020-06-12

**Authors:** Georgios Bizos, Efimia M. Papatheodorou, Theocharis Chatzistathis, Nikoletta Ntalli, Vassilis G. Aschonitis, Nikolaos Monokrousos

**Affiliations:** 1 Laboratory of Molecular Ecology, International Hellenic University, 57001 Thessaloniki, Greece; g.bizos@ihu.edu.gr; 2 Department of Ecology, School of Biology, Aristotle University, 54124 Thessaloniki, Greece; 3 Institute of Soil and Water Resources, Hellenic Agricultural Organization-Demeter, 57001 Thessaloniki, Greece; t.chatzistathis@swri.gr (T.C.); v.aschonitis@swri.gr (V.G.A.); 4 Department of Pesticides Control and Phytopharmacy, Benaki Phytopathological Institute, 8 S. Delta Str., 14561 Athens, Greece; nntali@agro.auth.gr

**Keywords:** abiotic stress, arbuscular mycorrhizal fungi, biocontrol agents, biofertilizers, endophytes, plant growth promoting rhizobacteria, transplantation stress

## Abstract

The olive tree (*Olea europaea* L.) is an emblematic, long-living fruit tree species of profound economic and environmental importance. This study is a literature review of articles published during the last 10 years about the role of beneficial microbes [Arbuscular Mycorrhizal Fungi (AMF), Plant Growth Promoting Rhizobacteria (PGPR), Plant Growth Promoting Fungi (PGPF), and Endophytes] on olive tree plant growth and productivity, pathogen control, and alleviation from abiotic stress. The majority of the studies examined the AMF effect using mostly *Rhizophagus irregularis* and *Glomus mosseae* species. These AMF species stimulate the root growth improving the resistance of olive plants to environmental and transplantation stresses. Among the PGPR, the nitrogen-fixing bacteria *Azospirillum* sp. and potassium- and phosphorous-solubilizing *Bacillus* sp. species were studied extensively. These PGPR species were combined with proper cultural practices and improved considerably olive plant’s growth. The endophytic bacterial species *Pseudomonas fluorescens* and *Bacillus* sp., as well as the fungal species *Trichoderma* sp. were identified as the most effective biocontrol agents against olive tree diseases (e.g., Verticillium wilt, root rot, and anthracnose).

## 1. Introduction

The olive tree (*Olea europaea* L.) is one of the most ancient cultivated crop species and the only one of the *Oleaceae* family that produces edible fruits [1]. It is an emblematic, long-living woody plant [2,3] of profound economic, societal, and environmental significance [4]. It is extensively cultivated in Mediterranean environments, and in several subtropical regions of South America, Australia, and Southern Africa. Currently, there is a worldwide increase in olive production due to the resilience of olive trees to climate change, and the growing demand for table olives and oil [5]. This demand is mostly generated by its tremendous nutritional value [6,7].

The olive plant can grow in very diverse environments [8] of soil pH (5.5–8.5) [1] and humidity, from arid to semi-arid regions [4] due to its high tolerance and high adaptability to poor soils, drought [9], salinity, and excess of boron and chlorine [1]. The enhancement of soil fertility and the fulfillment of its water requirements are crucial factors to attain high productivity [10]. The olive tree is widely known for its strong tendency for alternate bearing, with higher yields being produced every second year. Low soil fertility and many abiotic stresses, such as high temperatures and drought enhance this tendency [11]. Several abiotic stresses, especially water stress, are strongly related to the transplantation shock that occurs when olive cuttings or seedlings are transplanted in the field decreasing their survival rate [12]; this failure of the young olive plants to root well forces them to expend additional energetic cost in order to adapt to the new environmental conditions [13]. Meddad-Hamza et al. [14] showed that when young olive plants, with low root to shoot ratio, were transplanted into natural soil conditions without fertilizers, there was an abrupt decrease of the mineral uptake by the plant, causing stunted growth and increased plant mortality. Furthermore, the intensification in agricultural practices of olive cultivation, like the establishment of orchards with high tree densities for increasing olive production (in countries such as Spain, Italy, or Greece) caused the increase in both incidence and severity of olive pests and soil-borne diseases [1]. *Verticillium dahliae* and other soil borne pathogenic fungi like *Fusarium oxysporum*, *F. solani*, *Rhizoctonia solani*, and *Pythium* sp. are responsible for root rot and wilt diseases, with the wilting disease caused by *Verticillium dahliae* being the most severe disease of olive trees worldwide [15]. Olive knot disease caused by the Gram-negative bacterium *Pseudomonas savastanoi* pv. *savastanoi* is another common and important disease that infects the aerial parts of olive plant and causes severe damage and extensive production losses [16]. Similar implications may also occur due to Anthracnose disease, caused by the pathogenic fungi *Colletotrichum acutatum* and *Colletotrichum gloeosporioides*. It infects several parts of the olive tree such as flowers, leaves, shoots and fruits leading to severe yield losses. It also affects oil quality by increasing oil’s free acidity and peroxide level and deteriorates its organoleptic properties [17].

For these reasons, every year a large portion of the farmers’ revenue is spent on agrochemicals to promote olive trees’ growth, to control plant pathogens, and to increase the nutritional value and quality of the olive products. The public concern on the use of agrochemicals (i.e., chemically-based fertilizers, fungicides, insecticides, etc.) has increased in recent years, due to the negative effects on the environment (e.g., soil and groundwater pollution), the ecosystem’s biodiversity, and human and animal health [18,19]. The widespread use of fungicides or bactericides can lead to resistance increase of pathogenic fungi and bacteria to these chemicals and to the decrease of their efficacy, which in turn leads to their increasing use in order to maintain the same level of protection [20].

Consequently, several researchers have focused their efforts on the development of alternative, eco-friendly practices aiming to increase olive crop yield, to control biotic threats, and to ameliorate olive tree’s health [18]. An essential aspect of sustainable agricultural management is the development and implementation of eco-friendly methods and strategies that promote a soil’s biological processes, decrease agricultural inputs [21], and improve soil structure and fertility [22]. Towards this aim, microorganisms of plant’s rhizosphere could play a crucial role in plant health and growth [23]. Such microorganisms are the Arbuscular Mycorrhizal Fungi (AMF), the Plant Growth Promoting Rhizobacteria (PGPR), the Plant Growth Promoting Fungi (PGPF), and the Endophytes. The olive plant forms a strong relationship with these beneficial soil microbes, which enables the plant to grow not only in limiting nutrient soils but also under various abiotic stresses, such as water scarcity [6,9].

The objective of this study is to provide a review based on a literature survey of articles published during the last 10 years about the role of beneficial microorganisms (AMF, PGPR, PGPF, and Endophytes) as microbial inoculants on olive trees for supporting their growth, for their alleviation from abiotic and transplantation stresses and for the biological control of olive plant diseases.

## 2. Categorization of Studies

The search of the relevant literature published during the last 10 years (2009–March 2020) was conducted using reliable scientific databases of peer-reviewed, academic literature such as Scopus, Web of Science, and Google scholar. Overall, 67 research papers were identified that studied the effect of microbial inoculants on the olive tree’s growth and nutrition, quality of olive products, several types of abiotic stress, and olive plant diseases (Table S1). The keywords used in our search were: (“Arbuscular Mycorrhizal Fungi” or “AM fungi” or “AMF” or “Plant Growth Promoting Rhizobacteria” or “Promoting Rhizobacteria” or “PGPR” or “Plant Growth Promoting Fungi” or “Promoting Fungi” or “PGPF” or “Endophytes” or “endophytic microorganisms”) and (“olive” or “*Olea europaea*”) and (“growth” or “productivity” or “nutrition” or “water stress” or “drought” or “transplantation stress” or “salinity” or “saline stress” or “toxicity” or “biocontrol”). The results of our search were used to create our manuscript’s tables illustrating which microbial inoculants were used in olive tree studies over the last 10 years.

AMF and PGPR were the main subject of study (28 and 17 studies, respectively), followed by endophytes (nine), PGPF (six), and co-inoculants (seven). Furthermore, 27 studies examined the effect of microbial inoculants on biocontrol efficacy (biotic stress), 22 on olive tree growth, physiology, and crop productivity, 15 studies on the alleviation from abiotic stresses, and three on the alleviation from transplantation stress. The majority of the research studies were conducted in countries located in the Mediterranean basin (57), while the rest were conducted in South America (10). In North African countries such as Tunisia, Egypt, Morocco, and Algeria, where dry and calcareous soils are prevalent, 12, nine, six, and three studies were identified, respectively. These studies were mainly focused on increasing olive tree’s productivity in these types of soils. On the contrary, in the southern European countries such as Spain, Italy, and Greece (18, three, and two studies, respectively), as well as in Turkey (two studies), where olive cultivation is intensified, the studies mainly focused on the use of microbial inoculants as biocontrol agents against olive tree diseases.

## 3. Beneficial Microorganisms and Olive Tree’s Growth, Physiology, and Productivity

Most of the experimental studies focused on the beneficial role of AMF inoculation alone or with other microbes on olive trees’ growth, physiology, and productivity (Table 1). In these experiments, the *Glomus* species, and especially *Rhizophagus irregularis*, were the most frequently studied. In the mutual symbiosis with young olive plants, *Glomus* sp., *Glomus mosseae*, and *Glomus clarum* favored the augmentation of shoot and root biomass [6,24,25,26]. Similar observations were also made for *Gigaspora rosea* and *Scutelospora scrobiculata* [25]. *Glomus* sp. favored mineral nutrition and *G. Mosseae* activated olive plant’s defense system, by inducing the exchange of photosynthates and favoring the increase of phenol content [6,24]. According to Seifi et al. [6], the leaf chlorophyll content was higher when the olive plantlets were inoculated with *G. mosseae* or *R. irregularis*. Chenchouni et al. [26] demonstrated that a local AMF strain of *Glomus* sp. and a commercial strain of *R. irregularis* were the most effective ones on olive plantlet’s growth traits, in comparison to *Funneliformis mosseae*, *Septoglomus constrictum*, and *Gigaspora margarita*.

*R. irregularis* showed a higher percentage of root colonization compared to other species and it was more effective than *Glomus* sp. in promoting the young olive plant’s biomass [6,24,26]. It improved the nutritional status of olive plant, by influencing the increase of carbohydrate contents in the root, and by enhancing of P, K, Ca, Mg, Mn, Fe, and Zn uptake [27,28]. Sugar analysis of the olive roots showed that fructose and sucrose content was higher in the inoculated plants with *R. irregularis*, while glucose content was not affected [28,29]. The increased content of phenols and flavonoids in young olive plant roots is immensely significant in the plant-microbe symbiosis and they act as antimicrobial and antifungal plant defense agents [6,29]. The increased sink strength of mycorrhizal roots leads to enhanced translocation of sugars from source to leaves, which enables higher photosynthetic rates [28].

The effectiveness of an AMF species is even greater when it is co-inoculated with another AMF species. The co-inoculation of *R. irregularis* and *Glomus* sp. was more effective than *Glomus* sp. alone in promoting the increase in olive’s shoot biomass [24]. *R. irregularis* in combination with *G. mosseae* favors the increase of young olive plant height, root, and shoot biomass and improves P and K content [30]. Additionally, Chatzistathis et al. [31] found that AMF species (*Glomus* sp. and *Gigaspora* sp.) increased young olive plant growth and nutrient uptake in three olive genotypes. Furthermore, a mycorrhizal consortium of *Glomus*, *Acaulospora*, *Gigaspora*, *Entrophospora*, and *Scutellospora* species, increased the Na, Ca, and P concentrations in nutrient contents of ‘Haouzia’ olive cultivars under sandy loam soil conditions [32] and it was responsible for young olive plant-growth enhancement [12,32,33].

As regards PGPR inoculation studies, *Bacillus*, *Azospirillum,* and *Pseudomonas*, were the most commonly used genera (Table 1). Abd-Alhamid et al. [22] reported that the reduced application of nitrogen fertilization (75%) in combination with three bacteria species (*Azotobacter chrococcum*, *B. megaterium,* and *B. circulans*) increased the yield and several fruit physical parameters (e.g., weight, volume, length and diameter, fruit oil content, oil quality, and total microbial count) in comparison to the standard orchard practice. Haggag and Merwad [34] showed that the reduction of the recommended dose of mineral fertilization (N, P, K) by 50%, combined with three biofertilizer products, containing *Azospirillum* sp., *Bacillus mucilaginosus,* and *Bacillus megatherium*, increased the young plant’s root system. The bacterial strains *Pseudomonas fluorescent* P19 or P21 [35] *Chryseobacterium* sp. AG13, *Chryseobacterium* sp. CT348, *Pseudomonas* sp. CT364 and *Azospirillum brasilense* Cd [36,37] promoted root induction and formation to a similar or greater extent than the control plants treated with indole-3-butyric acid, due to their ability to synthesize at low rates and secrete continually indole-3-acetic acid (IAA) [35,36].

Ramasamy et al. [38] stated that PGPR and AMF co-inoculation can be more beneficial for plant growth and nutrient uptake than a single inoculation, probably because PGPR can enhance the AMF establishment, by stimulating the hyphal growth through the production of vitamins and enzymes or by increasing the permeability of root epidermis cell wall [39]. The reduction of the recommended dose of mineral fertilization (N, P, K) by 50% to 70% , combined with a mixed inoculant containing PGPR (*Azotobacter chroococcum*, *Azospirillum brasilense*, *Bacillus megaterium* var. *phosphaticum*, *Bacillus cereus*, *Pseudomonas* sp.) and AMF strains (*G. mosseae* NRC31 *Glomus fasciculatum* NRC15) stimulated plant growth and enhanced the nutritional status of the olive seedlings [10]. Costa and Melloni [40] also showed that the co-inoculation of PGPR (*Pseudomonas* sp. and *Paenibacillus* sp.) with AMF species (*Acaulospora scrobiculata*, *Gigaspora rosea*, and *Rhizophagus clarus*) could effectively promote the nutritional status and growth of young olive plants. Furthermore, Shaheen and Taweel [21] showed that the reduction of the standard dose of mineral fertilization by 50% in combination with *Bacillus polymyxa* and fungus yeast *Saccharomyces cerevicisiae* foliar co-inoculation, increased significantly olive fruit length, weight, and width. The foliar application of *S. cerevicisiae* combined with AMF root colonization, also showed higher values of leaf N, K, Fe, and Mn content and higher values of auxin and gibberellin contents in these treatments, compared to un-inoculated control plants [41].

The aforementioned studies showed that the use of inoculants with multiple AMF species was more efficient for promoting plant biomass and productivity than individual species’ use. In the case of PGPR and AMF, their effects were examined in cases where inorganic fertilizers were also applied, but further studies without fertilizers were recommended by the authors in order to test whether they are also effective in nutrient-limited soils.

## 4. Beneficial Microbes and a Plant’s Response to Abiotic and Transplantation Stress

Inoculation with AMF species was also the focus of studies that investigated plant alleviation from abiotic and transplantation stresses. Once again, the most frequently beneficial AMF species used, under abiotic and transplantation stress, were *Glomus* sp. and *R. irregularis* (Table 2). *Glomus claroideum* and *G. mosseae* reduced the detrimental effects of salinity and strengthened the young olive plant’s capacity to resist water stress [14,42]. *G. mosseae* favored K acquisition by plants, which plays a major role in regulating the osmotic processes reducing significantly the saline stress [42]. It stimulated more the root growth than the shoot growth, where the higher root/shoot ratio causes better hydro-mineral nutrition, reinforcing the capacity of the young olive plants to resist not only water stress [41] but also transplantation stress [43]. *R. irregularis* was superior to *G. mosseae* in terms of root colonization on young olive plant roots under saline stressed conditions [44]. *R. irregularis* favors K uptake by the plants and reduces the effects of saline stress but not to the same extent as *G. mosseae* [42]. In addition, the co-inoculation with two *R. irregularis* strains (GC2 and GA5) also protected the olive plant from saline stress by decreasing the malondialdehyde content and by activating the antioxidant defenses of the plant [13,45]. M’barki et al. [46] showed clearly that chlorophyll and carotenoid contents (essential for photosynthesis) were significantly enhanced when the roots were colonized by *R. irregularis*. The higher level of root colonization by *R. irregularis* leads to the creation of an ample hyphal network in the rhizosphere, thus establishing better transport of nutrients to the plant [14]. *R. irregularis* favored root development when olive plantlets were grown in phosphorus poor soils, while in P rich soils, the inoculation with AMF resulted in reduced shoot growth compared to untreated control plants [47]. The olive plant has low phosphorus requirements and the excess of P can have adverse effects, such as a reduction of shoot growth and a possible reduction of AMF colonization [47].

In soils with high levels of gypsum, the mycelial hyphae of *R. irregularis* mitigated the ascent of the toxic gypsum sulfate ions from the roots to the shoots [48]. Furthermore, Briccoli Bati et al. [49] demonstrated that a microbial inoculant containing AMF species (*Glomus* sp., *Scutellospora heterogama*, *Paraglomus laccatum*, and *Diversispora celata*) instigated a protective effect against heavy metal toxicity caused by high soil Mn concentration. The major part of Mn was retained in the fungal and spore cell walls, bound to components such as chitin, cellulose, and cellulose derivatives.

The extra-radical hyphae created by the inoculation with six different *Glomus* species (*G. etunicatum*, *G. microaggregatum*, *G. geosporum*, *G. claroideum*, *G mosseae*, and *R. irregularis*) favored the ability of the root to expand in a larger volume of soil and to regulate stomatal conductance and root hydraulic conductivity, thus improving the gas exchanges and water relation status of the young olive plants [4,50]. Ouledali et al. [4] suggested that stomata closure in inoculated olive plants may be mediated by a putative mycorrhiza-dependent metabolite replacing the phytohormone abscisic acid, which controls stomatal opening. This metabolite may be produced by the fungus itself or by the plant. The accumulation of this metabolite is a result of the AM fungal symbiosis [4] and supports plants’ growth under drought conditions.

PGPRs can also be used in the alleviation of abiotic stresses, such as increased soil calcicity, which is frequently encountered in the Mediterranean region [19], and they can operate optimally in a wide spectrum of temperatures and soil pH [51]. The bacterial strains *Azospirillum brasilense* BR11001t, *Azospirillum amazonense* BR11040t, *Herbaspirillum seropedicae* BR11175t, and *Burkholderia brasilensis* BR11340t favored root induction in a wide range of pH (5–9) and temperature (15–35 °C) conditions [51]. The application of *Bacillus megatherium* along with antioxidants (e.g., ascorbic and citric acid) improved olive yield, fruit weight, and flesh oil content in calcareous soils. This was attributed to *B. megatherium*’s phosphate solubility and the antioxidant auxinic action that improved the P level of the olive plants [52]. The inoculation with *Azotobacter chroococcum* had a similar effect on the olive plant in calcareous soils, where the bacteria improved biomass production through the production of phytohormones and enhanced transfer of essential nutrients like N, P, and K from soil to plant [19].

El-Shazly and Ghieth [53] demonstrated that the interaction of PGPR (*Azotobacter chroococcum*) and AMF (*Glomus macrocarbium*), combined with the application of humic acid, reduced the negative impact of salinity. The combined treatment showed higher values of total microbial counts, bacterial densities, AMF infection percentage, and soil enzymatic activity. The study suggested that the production of phytohormones such as IAA, gibberellins, cytokinins and ethylene, nitrogen fixation, phosphates, and nutrient solubilization was the cause for these increases.

The majority of the aforementioned studies, examined the ability of the microbial inoculants to support the growth of olive plants in abiotically stressed environments (e.g., in saline soils, in calcareous soils, in soils with an excess of heavy metals or in drought conditions). In most cases, the studies investigated the combined inoculation of AMF and PGPR strains, applied either alone or together with other constituents, such as antioxidants or humid acids.

## 5. Beneficial Microorganisms Against Pathogens

Over the last decade, PGPR (10 studies), endophytes (nine), and PGPF (six) have been the center of attention for their biocontrol potential against major olive plant diseases, whereas only two studies with AMF were conducted for this purpose (Table 3). Many strains of *Bacillus* and *Pseudomonas* have been used in agriculture as biocontrol agents against several diseases caused either by fungi or bacteria [54]. Sanei [55] reported that eight isolates of *Pseudomonas fluorescens* inhibited *Verticillium*’s microsclerotia germination. The long-term endurance of microsclerotia and the fact that they cause the initial infections, make them direct targets of biocontrol of *Verticillium dahliae* [15,55]. Furthermore, Markakis et al. [56] showed that the application of *Paenibacillus alvei* K165 was also associated with the reduction of microsclerotia germination. The application of this bacterial strain in ‘Kalamon’ cultivar (highly tolerant to *V. dahliae*) was very effective against Verticillium wilt. Gómez-Lama Cabanás et al. [23] reported that three *Pseudomonas* strains (PIC25, PIC105, and PICF141) and three novel bacteria strains *Paenibacillus polymyxa*, *Paenibacillus terrae,* and *Bacillus* sp. PIC28 [15] demonstrated effectiveness against the highly virulent *V. dahliae* isolate (D pathotype), with the strain PICF141 being the most effective. Their biocontrol abilities rely on the production of cell wall-degrading enzymes (protease, cellulase, phytase amylase, etc.), siderophores, indole acetic acid, and ammonia [23].

Strains of *Bacillus subtilis* were very effective against Verticillium wilt, root rot, olive knot, and anthracnose [17,54,57]. *B. subtilis* Y1336 strain significantly reduced the disease incidence of *V. dahliae* and other soil-borne diseases like *Fusarium oxysporum, Fusarium solani*, *Rhizoctonia solani*, and *Pythium* sp. [57]. *B. subtilis* 2515-1 strain decreased the tumors in olive’s stems caused by *Pseudomonas savastanoi pv. Savastanoi* 2064-8 strain [54]. *B. subtilis* QST 713 strain significantly reduced the percentage of latent infections of anthracnose on olive fruits, caused by *Colletotrichum acutatum* and *Colletotrichum gloeosporioides* [17]. The effectiveness of these strains relies on the ability of *Bacillus* species to promote growth by increasing nutrient availability [57] and on their high, antibacterial specificity with *Pseudomonas savastanoi* pv. *savastanoi* strains [54]. They can also be used as an alternative to chemical or other Cu-based products that are mainly used to treat the symptoms of anthracnose [17].

Co-inoculation with bacterial strains *Azotobacter* sp. AZM1, *Bacillus cereus* BCM8, *B. megaterium* BMM5, and *B. subtilis* BSM1 had positive results against *F. oxysporum*, *F. solani*, and *R. solani* in olive transplants [58], suggesting that such consortiums should be further investigated in the future. Root rot caused by *Fusarium solani* and *oxysporum* can be greatly reduced by *Bacillus licheniformis* and *Enterobacter colcae* in combination with carbendazim (fungicide), suggesting that this can be an alternative method to the use of synthetic fungicide alone, reducing the risk of the occurrence of fungicide resistance and its harmful impact to the environment [59].

The endophytic *Pseudomonas fluorescens* PICF7 strain was the most frequently used microorganism as biocontrol agent in olive trees (Table 3). *P. fluorescens* PICF7 can penetrate (through root hairs) and colonize olive root tissues and trigger a wide array of defense responses, both local (roots) and systemic (aerial tissues), which explain its biocontrol effectiveness against diseases such as Verticillium wilt [2,60,61]. The effective application of this endophyte requires its presence in both the surface and the interior of olive roots before colonization by *V. dahliae* and eventually its direct contact with the pathogen [62,63]. Apart from *P. fluorescens* PICF7, another *Pseudomonas* strain that is highly competent in colonizing the rhizosphere and able to suppress the deleterious effects of Verticillium wilt is the *Pseudomonas putida* PICP2 strain [2]. In the case of anthracnose, which affects the flowers and the fruits of the olive plant, a spray application at the blooming stage of three endophytic isolates of yeast-like fungus *Aureobasidium pullulans* (well adapted in the phyllosphere), significantly reduced the percentage of latent infections of the disease compared to the untreated control [17]. These researchers suggested that the endophyte should be applied at the blooming stage because it can colonize living plant tissues and produce antifungal and antibacterial compounds.

Furthermore, two very promising candidates with biocontrol abilities are the endophytic *Bacillus* strains, *B. licheniformis* LMRE 36 [64] and *B. velezensis* OEE1 [65]. *Bacillus licheniformis* LMRE 36 strain inhibited *Fusarium solani* strains Fso6 and Fso7 progression up to 75% compared to the untreated plants suggesting that secondary metabolites from *Bacillus* strongly interfere with fungal pathogens [64]. *B. velezensis* OEE1 strain was more effective in reducing the disease severity of *Fusarium Solani* compared to commercial fungicides [65]. These beneficial effects could be explained by phosphate solubilization, nitrogen fixation, and IAA production that are attributed to *B. velezensis* OEE1 endophytic lifestyle.

*Trichoderma harzianum* Ths97 strain was very effective against *Fusarium solani* strain Fso14, which is responsible for the root rot in olive plants [5]. *T. harzianum* produces antibiotics and hydrolytic enzymes that are usually regulated by jasmonic acid and ethylene dependent signaling pathway that triggers a variety of plant defense responses [5]. *Trichoderma asperellum* Bt3 and T25 strains delayed the disease outbreak and decreased the total severity of Verticillium wilt [66]. These researchers advocated that the successful root colonization by the *Trichoderma* strain is a major requirement for plant defense system. The difference of the root colonization was also influenced by the application method since *T. harzianum* CECT 2413 strain was unable to colonize the olive roots of ‘Picual’ cultivar when it was applied as (pregerminated) conidia suspensions [66,67]. Conidia can be highly sensitive to soil fungistasis and have a slow survival rate, especially under natural conditions. These limitations can be overcome by the use of cornmeal sand mixture soil medium [68]. *Trichoderma* species can be very effective when they are combined with field applications, such as solarization, as this combination decreased the microsclerotia of *Verticillium* [69].

Boutaj et al. [70] reported that *Glomus irregulare* was very effective at reducing the disease symptoms caused by Verticillium wilt. These authors suggested that the induction of systemic resistance caused by the accumulation of phenolic compounds and flavonoids in the roots is the main mechanism of disease. Co-inoculation with *Lactobacillus plantarum*, *Lactobacillus casei*, *Rhodobacter sphaeroides*, *Rhodopseudomonas palustris*, *Saccharomyces* sp., *Streptococcus lactis,* and *Streptomyces* sp. in combination with solid οlive oil waste compost (with urea as nitrogen source) was effective against the highly virulent *V. dahliae* isolate V024 and reduced the disease incidence to 80% [71]. Moreover, a co-inoculation of *T. harzianum* Rifai strain KRL-AG2 with several AM fungi (*R. irregularis*, *G. aggregatum*, *G. mosseae*, *G. clarum*, *G. monosporus*, *G. deserticola*, *G. brasilianum*, *G. etunicatum*, and *G. margarita*) also significantly decreased the Verticillium wilt severity in a field experiment [72]. Lastly, Mulero-Aparicio et al. [73] showed that Fusarium oxysporum FO12 strain significantly reduced significantly the disease incidence in young olive plants and adult trees.

Based on the aforementioned studies, several mechanisms were reported as crucial ones for the control of pathogens, especially for Verticillium wilt, which was the most studied disease in olive trees. PGPR acted mostly by inhibiting Verticillium’s microsclerotia germination, or by producing cell wall-degrading enzymes. AMF induced the accumulation of phenolic compounds and flavonoids in roots, making the plants less vulnerable to diseases, while endophytes induced plant defense mechanisms mainly related to phosphate solubilization, nitrogen fixation, and/or IAA production.

## 6. Inoculum-Cultivar Specificity

There is a strong interaction between the effectiveness of a microbial inoculant and the olive cultivar. The AMF genera may differently affect olive cultivars, growing under the same soil conditions, in terms of vegetative growth and nutritional status since mycorrhiza affect the root system morphology [31]. *Gigaspora* sp. colonized the root system of ‘Chondrolia Chalkidikis’, ‘Koroneiki’, and ‘Kothreiki’, while *Glomus* sp. colonized only the root system of ‘Koroneiki’ [31]. ‘Chemlaly’, ‘Quartina’, and ‘Coronaki’ olive cultivars showed different responses to vegetative growth and leaf nutrient content when they were inoculated by the same fungi [41]. In this context, ‘Koroneiki’ demonstrated higher AMF colonization rate compared to ‘Valanolia’, which led to higher growth attributes in ‘Koroneiki’ (higher height and a higher number of lateral shoots and leaves, as well as higher oil phenol content); in contrast, ‘Valanolia’ showed higher leaf area [6]. The intensity of colonization depends on the cultivar and inoculum [49]. *Glomus clarum, Gigaspora rosea*, and *Scutelospora scrobiculata* stimulated plant growth in ‘Arbequina’ and ‘Grappolo’ cultivars, whereas ‘Maria da Fé’ variety had low mycorrhizal dependency [25]. The same bacterial strains induced rooting in ‘Hojiblanca’ but not in ‘Arbequina’ and ‘Picual’ [36]. The same combined treatment (PGPR and AMF) applied on ‘Arbequina’ and ‘Maria da Fé’ cultivars promoted high growth (height and diameter), fresh root, and shoot dry matter production in ‘Arbequina’ and improvement of nutritional status (higher nitrogen contents in the shoot) in ‘Maria da Fé’ [40]. Boutaj et al. [32], who investigated the inoculation of the olive cultivar ‘Picholine Marocaine’ with an autochthonous mycorrhizal inoculum, and with the commercial pure strain *Glomus irregulare*, found that root colonization and mycorrhizal frequency and intensity were greatly improved in both the autochthonous consortium (100% and 58.91%, respectively) and *Glomus irregulare* (97.77% and 57.77%, respectively) compared to the control plants.

From all the above quoted information, it is clear that the inoculum-olive cultivar specificity plays a crucial role not only on boosting plant growth, modifying root morphology, and enhancing plant nutrition, but also on improving the qualitative characteristics of olive products (e.g., high polyphenol olive oil). Thus, more emphasis should be given in the near future on the optimum interaction among soil and cultivation management techniques (which influence soil microbiology and AMF abundance) and choice of suitable genotype(s) (showing high qualitative characteristics) to maximize the benefits for the consumers’ health. Furthermore, because of the idiosyncratic interactions between olive cultivar and AMF, PGPR, or endophytes’ species, it is suggested to use a mixed inoculum, where various microbial species/strains of the above categories could be applied simultaneously. In that case, the cultivar has the chance to interact with the most successful microbial species to overcome difficulties in growth.

## 7. Microbial Inoculation under Nursery and Field Conditions

The microbial inoculation in the nurseries before their transplantation into the field is a beneficial strategy for olive plant growth promoting its resistance against biotic and abiotic stresses [12,42]. Growing young plants in nurseries, and applying the experimental inoculants under totally controlled soil conditions offers a great advantage in terms of root colonization. This literature review showed that most of the AMF experiments were conducted in nurseries under either unstressed or stressed conditions. An early mycorrhizal inoculation of olive seedlings during nursery propagation can be extremely beneficial for the early plant establishment and crop productivity, as commercial yields would be reached earlier [6,29,30]. Jiménez-Moreno et al. [47] showed that it is much more advantageous to apply the AMF inoculum in the nursery than directly in the cultivation field, due to the increased root volume of the plant before transplantation. The benefit of AMF inoculation at the early plant stages can be effective for a long time (in most cases at least four years after transplantation, expressed with a high commercial yield) [44]. Furthermore, AMF inoculation in the nursery can reduce the harmful effect of Verticillium wilt and can produce more vigorous plants with enhanced resistance against the pathogen [70]. This is associated to the competition for space on plants’ roots between the pathogen and the AMF; the AMF occupy the root surface area, and do not allow the pathogen to infest it, thus reducing the occurrence of the symptoms caused by the soil-borne pathogens in the field.

The effect of biocontrol agents against olive plant diseases has rarely been investigated under field conditions. The application of microbial biocontrol agents against olive plant pathogens, at the propagation stage (greenhouse or nursery) is once again the most recommended approach, not only because it is an effective, preventive strategy (by transferring into the field the plants with the defense mechanisms ‘activated’ and/or harboring a cohort of effective antagonists), but also because the use of biological control agents under these conditions is easier, cheaper, and less time-consuming than large-scale field treatments [15]. Hibar et al. [57] suggested that an earlier treatment (in the nursery) with biofungicide can significantly reduce the occurrence of symptoms caused by soil borne-pathogens in the field.

Some studies tried to emulate in the nursery the field conditions by using various soil mediums, with the cornmeal sand mixture medium resulting to be the best [67,68]. Even though this approach was characterized successful in terms of emulating the soil conditions, the researchers acknowledged that the natural field conditions were difficult to be replicated as the infestation speed differed considerably.

## 8. Future Perspectives

In order to evaluate thoroughly the efficacy of a biological control agent, more field experiments should be conducted, which is the last stage of a BCA’s efficacy assessment, because the pathogenic inoculum density usually recorded in the field is very difficult to be replicated in a controlled nursery experiment. Moreover, more studies should be conducted assessing the biocontrol efficacy of AMF against olive plant diseases, as well as to examine the effectiveness of biocontrol agents that combine AMF species with other microbial inoculants, such endophytes, against olive plant diseases that infect the aerial part of the plant. Nevertheless, the most important challenge would be the assessment of the microbial inoculants’ influence on the phenolic content of olive products, which is among the most important qualitative characteristics of extra virgin olive oil. Finally, our search showed that several combined AMF, PGPR, or endophyte inoculant consortia showed promising results and they should be considered for further production by the biofertilizer industry.

## 9. Conclusions

Based on the literature review of the last 10 years, it was observed that the majority of the studies in *Olea europaea* L. focused on AMF inoculation, due to its high ability to stimulate olive plant’s growth and nutritional status, ameliorate its antioxidant capacity, and reduce the impact of abiotic and biotic stresses. Nevertheless, this efficacy is strongly correlated to the AMF genera and the type of cultivar. *R. irregularis* and *G. mosseae,* mainly in co-inoculants, were the most effective agents. *Glomus irregulare* was also very effective against Verticillium wilt. Furthermore, the impact of AMF inoculation is greater in the first stages of plant development. Regarding PGPR, the majority of the microbial inoculants contained nitrogen-fixing bacteria (e.g., *Azospirillum* sp.) and potassium or phosphorous solubilizing bacteria (mainly *Bacillus* species). These microbial inoculants combined with mineral fertilizers or manure supply were very effective in enhancing olive plants’ growth and with less agricultural inputs.

As regards the studies on biotic stress, most of them were focused on bacteria, endophytes, and fungi that belong to *Pseudomonas*, *Bacillus*, and *Trichoderma* species. Bacterial, endophytic strain *Pseudomonas fluorescens* PICF7 was the most studied and the most effective biocontrol agent against Verticillium wilt. It was more effective when it was present in both the surface and the interior of olive organs and in direct contact with the pathogen. The application of biological control in olive plants, at the propagation stage, is the most recommended approach because an earlier treatment (in the nursery) with bio-fungicide can significantly reduce the occurrence of symptoms caused by pathogens in the field.

## Figures and Tables

**Table 1:** Microbial inoculant applied to improve olive tree’s growth, physiology, and crop productivity. AMF: Arbuscular Mycorrhizal Fungi; PGPR: Plant Growth Promoting Rhizobacteria.

Microbial Inoculant	Type	Product	Effect	Application	Age	Location	References
*Rhizophagus irregularis, Glomus mosseae*	AMF	-	Growth	Root	Young	Nursery	[6]
*R. irregularis*	AMF	-	Growth and Nutrition *	Root	Young	Nursery	[7]
*Glomus* sp., *Acaulospora* sp., *Glomus macrocarpum, Glomus multicaulis*, *Scutellospora* sp.	AMF	-	Growth	Root	Young	Nursery	[12]
*Glomus* sp., *R. irregularis*	AMF	-	Growth	Root	Young	Experimental Field	[24]
*Glomus clarum, Gigaspora rosea*, *Scutellospora scrobiculata*	AMF	-	Growth	Root	Young	Nursery	[25]
*Glomus* sp., *Septoglomus constrictum*, *R. irregularis, Funneliformis mosseae*, *Gigaspora margarita*	AMF	-	Growth	Root	Young	Nursery	[26]
*R. irregularis*	AMF	-	Growth and Nutrition	Root	Young	Nursery	[27]
*R. irregularis*	AMF	-	Growth and Nutrition **	Root	Young	Nursery	[28]
*R. irregularis*	AMF	-	Growth and Nutrition **	Root	Young	Nursery	[29]
*R. irregularis, G. mosseae*	AMF	-	Growth and Nutrition	Root	Young	Nursery	[30]
*Glomus* sp., *Gigaspora* sp.	AMF	-	Growth and Nutrition	Root	Young	Nursery	[31]
*Glomus* sp., *Acaulospora* sp., *Gigaspora* sp., *Entrophospora* sp., *Scutellospora* sp., *Glomus irregulare* DAOM 197198	AMF	-	Growth and Nutrition	Root	Young	Nursery	[32]
*Acaulospora scrobiculata*, *G. macrocarpum, R. irregularis*, *Glomus versiforme, Gigaspora* sp., *Scutellospora fulgida, Glomus geosporum*, *Glomus aureum, Glomus microcarpum*, *Glomus aurantium, G. corymbiforme*, *G. clarum, Scutellospora heterogama*	AMF	-	Growth	Root	Young	Nursery	[33]
*Azotobacter chrococcum*, *Bacillus megaterium, Bacillus circulans*	PGPR	-	Growth	Root	Adult	Orchard	[22]
*Azospirillum* sp., *Bacillus mucilaginosus*, *B. megatherium*	PGPR	-	Growth and Nutrition	Root	Young	Nursery	[34]
*Pseudomonas fluorescent* P19, P21	PGPR	-	Growth	Root	Young	Nursery	[35]
*Pantoea* sp. AG9, *Chryseobacterium* sp. AG13, *Pseudomonas* sp. CT364, *Chryseobacterium* sp. *CT348*, *Azospirillum brasilense* Cd	PGPR	-	Growth	Root	Young	Nursery	[36]
*Azospirillum brasilense*	PGPR	Azototal^®^	Growth	Root	Young	Nursery	[37]
*Azotobacter chroococcum*, *Azospirillum brasilense*, *B. megaterium* var. *phosphaticum*, *G. mosseae* NRC31, *Pseudomonas* sp., *Bacillus cereus*, *Glomus fasciculatum* NRC15	AMF and PGPR	-	Growth and Nutrition	Root	Young	Nursery	[10]
*Pseudomonas* sp., *Paenibacillus* sp., *Acaulospora scrobiculata*, *Gigaspora rosea, Rhizophagus clarus*	AMF and PGPR	-	Growth and Nutrition	Root	Young	Nursery	[40]
*Bacillus polymyxa*, *Saccharomyces cereivisea*	PGPR and Fungi	Biovine^®^	Growth and Nutrition	Foliar	Adult	Orchard	[21]
AMF consortium, *S. cerevisiae*	AMF and Fungi	-	Growth and Nutrition	Root/Foliar	Young	Nursery	[41]

* Enhancement of the nutritional value of the olive fruit with the increase of antioxidants with health benefits ** Enhancement of the antioxidant capacity of the olive tree with the increase of phenols and flavonoids.

**Table 2:** Microbial inoculant studies aiming on the alleviation of abiotic and transplantation stresses.

Microbial Inoculant	Type	Product	Stress Conditions	Application	Age	Location	References
*G. etunicatum, G. microaggregatum*, *R. irregularis, G. claroideum*, *G. mosseae, G. geosporum*	AMF	Symbivit^®^	Water Stress	Root	Young	Experimental Field	[4]
*Glomus* sp., *R. irregularis*, *Dominikia* sp*., Funneliformis* sp., *Funneliformis geosporum, Septoglomus constrictum, Septoglomus viscosum*	AMF	-	Water Stress	Root	Young	Nursery	[8]
*Rhizophagus manihotis*, *Funneliformis mosseae*	AMF	-	Water Stress	Root	Young	Nursery	[9]
*R. irregularis* GC2, GA5	AMF	-	Transplantation	Root	Young	Nursery	[13]
*G. mosseae, R. irregularis*	AMF	-	Transplantation	Root	Young	Nursery	[14]
*G. mosseae, R. irregularis, G. claroideum*	AMF	-	Salinity	Root	Young	Nursery	[42]
*R. irregularis, G. trimurales, G. manihoti, G. diaphanum, G. pansihalos*, *G. geosporum, G. glomerulatum*, *G. fasciculatum, G. etunicatum*, *Acaulospora* sp., *Acaulospora mellea*, *Entrophospora kentinensis*, *Scutellospora nigra*, *Scutellospora fulgida*, *Scutellospora heterogama,*	AMF	-	Transplantation	Root	Young	Nursery	[43]
*R. irregularis, G. mosseae*	AMF	-	Salinity	Root	Young	Experimental Field	[44]
*R. irregularis* GC2, GA5	AMF	-	Water Stress	Root	Young	Nursery	[45]
*R. irregularis*	AMF	-	Water Stress	Root	Young	Nursery	[46]
*R. irregularis*	AMF	-	Nutrient stress	Root	Young	Nursery	[47]
*R. irregularis*	AMF	-	Toxicity	Root	Young	Nursery	[48]
*Glomus* sp., *Scutellospora heterogama, Paraglomus laccatum, Diversispora celata*	AMF	Endorize IV^®^	Toxicity	Root	Young	Nursery	[49]
*R. irregularis, G. etunicatum*, *G. microaggregatum, G. claroideum*, *G. mosseae, G. geosporum*	AMF	Symbivit^®^	Water Stress	Root	Young	Nursery	[50]
*Azotobacter chrococcum*	PGPR	-	Toxicity	Root	Young	Nursery	[19]
*Azospirillum brasilense* BR11001t, *Azospirillum amazonense* BR11040t*, Herbaspirillum seropedicae* BR11175t, *Burkholderia brasiliensis* BR11340t	PGPR	-	Temperature and pH	Root	Young	Nursery	[51]
*B. megatherium*	PGPR	Phosphorine^®^	Toxicity	Root	Young	Nursery	[52]
*Azotobacter chroococcum*, *G. macrocarbium*	PGPR and AMF	-	Salinity	Root	Young	Experimental Field	[53]

**Table 3:** Microbial inoculants used as biocontrol agents. PGPF: Plant Growth Promoting Fungi.

Microbial Inoculant	Type	Product	Disease	Application	Age	Location	References
*Pseudomonas fluorescens* PICF7	Endophyte	-	Verticillium wilt	Root	Young	Nursery	[2]
*P. fluorescens* PICF7	Endophyte	-	Verticillium wilt	Root	Young	Nursery	[3]
*Trichoderma harzianum* Ths97	PGPF	-	Root rot	Root	Young	Nursery	[5]
*Paenibacillus polymyxa, Paenibacillus terrae, Bacillus* sp. PIC28, *P. fluorescens*PICF7, *Pseudomonas* sp. PICF141	PGPR	-	Verticillium wilt	Root	Young	Nursery	[15]
*Bacillus mojavensis* A-BC-7	PGPR	-	Olive knot	Stem	Young	Nursery	[16]
*B. subtilis* QST 713*, Aureobasidium pullulans* A3, A5, A6	PGPR Endophyte	Serenade Max^®^	Anthracnose	Foliar	Adult	Orchard	[17]
*Pseudomonas* spp. PIC25, PIC105, PICF141	PGPR	-	Verticillium wilt	Root	Young	Nursery	[23]
*R. irregularis, G. mosseae*	AMF	-	Verticillium wilt	Root	Young	Experimental Field	[44]
*B. subtilis* 2515-1	PGPR	-	Olive knot	Stem	Young	Nursery	[54]
*P. fluorescens*	PGPR	-	Verticillium wilt	Root	Young	Nursery	[55]
*Paenibacillus alvei* K165	PGPR	-	Verticillium wilt	Root	Young	Experimental Field	[56]
*B. subtilis* Y1336	PGPR	Biobac^®^	Root rot and wilt	Root	Young	Nursery	[57]
*Azotobacter* sp. AZM1, *B. cereus* BCM8, *B. megatherium* BMM5, *B. subtilis* BSM1	PGPR	-	Root rot	Root	Young	Nursery	[58]
*B. licheniformis, Enterobacter colcae*	PGPR	-	Root rot	Root	Young	Nursery	[59]
*P. fluorescens* PICF7, *P. putida* PICP2	Endophyte	-	Verticillium wilt	Root	Young	Nursery	[60]
*P. fluorescens* PICF7	Endophyte	-	Olive knot	Root	Young	Nursery	[61]
*P. fluorescens* PICF7	Endophyte	-	Verticillium wilt	Root	Young	Nursery	[62]
*P. fluorescens* PICF7	Endophyte	-	Verticillium wilt	Root	Young	Nursery	[63]
*B. licheniformis* LMRE 36	Endophyte	-	Root rot	Root	Young	Nursery	[64]
*B. velezensis* OEE1	Endophyte	-	Olive knot	Root	Young	Nursery	[65]
*Trichoderma asperellum* Bt3, T25	PGPF	-	Verticillium wilt	Root	Young	Nursery	[66]
*T. harzianum* CECT 2413	PGPF	-	Verticillium wilt	Root	Young	Nursery	[67]
*Fusarium oxysporum* FO12, *Phoma* sp. Ph02	PGPF	-	Verticillium wilt	Root	Young	Nursery	[68]
*T. harziarum*	PGPF	-	Verticillium wilt	Root	Adult	Orchard	[69]
*Glomus* sp., *Acaulospora* sp.*, Gigaspora* sp., *Entrophospora* sp., *Scutellospora* sp., *G. irregulare* DAOM 197198	AMF	-	Verticillium wilt	Root	Young	Nursery	[70]
*Fusarium oxysporum* FO12, *Lactobacillus plantarum, Lactobacillus casei, Rhodobacter sphaeroides, Rhodopseudomonas palustris Saccharomyces* sp., *Streptococcus lactis, Streptomyces* sp.	PGPR and Fungi	EM-1^®^	Verticillium wilt	Root	Young	Nursery	[71]
*G. aggregatum, G. mosseae*, *G. clarum, G. monosporus, G. deserticola*, *G. brasilianum, G. etunicatum*, *G. margarita*, *R. irregularis*, *T. harzianum* Rifai KRL-AG2	AMF and PGPF	T-22Planter Box^®^	Verticillium wilt	Root	Adult	Orchard	[72]
*Fusarium oxysporum* FO12	PGPF		Verticillium wilt	Root	Young/Adult	Nursery/Orchard	[73]

## Supplementary Materials

The following are available online at https://www.mdpi.com/2223-7747/9/6/743/s1, Table S1: List of all the research articles used in our study.
