# Supplementary material for: The Role of Microbial Inoculants on Plant Protection, Growth Stimulation, and Crop Productivity of the Olive Tree (Olea europea L.)
Source: Plants (Basel). 2020 Jun 12;9(6):743. doi: 10.3390/plants9060743 (PMC7356289; doi:10.3390/plants9060743)
Supplement: Supplementary file 1 [file plants-09-00743-s001.pdf]

Table S1. List of all the research articles used in our study

1. Abdel-Monaim, M.F.; EL-Morsi, M.E.A.; Hassan, M.A.E. Control of root rot and wilt disease complex of some evergreen fruit transplants by using plant growth promoting rhizobacteria in the New Valley Governorate , Egypt. **2014**, *1*, 23–33.

2. Abd-Alhamid, N.; Hassan, H.S.A.; Haggag, L.F.; Hassan, A.M. Effect of mineral and bio-fertilization on vegetative growth, leaf mineral contents and flowering of manzanillo olive trees. *Int. J. ChemTech Res.* **2015**, *8*, 51–61.

3. Alguacil, M. del M.; Torrecillas, E.; Kohler, J.; Roldàn, A. A molecular approach to ascertain the success of "in situ" AM fungi inoculation in the revegetation of a semiarid, degraded land. *Sci. Total Environ.* **2011**, *409*, 2874-2880.

4. Arici, S.E.; Demirtas, A.E. The effectiveness of rhizosphere microorganisms to control Verticillium wilt disease caused by *Verticillium dahliae* Kleb. in olives. *Arab. J. Geosci.* **2019**, *12*, 781.

5. Ben Amira, M.; Lopez, D.; Triki Mohamed, A.; Khouaja, A.; Chaar, H.; Fumanal, B.; Gousset-Dupont, A.; Bonhomme, L.; Label, P.; Goupil, P.; et al. Beneficial effect of *Trichoderma harzianum* strain Ths97 in biocontrolling *Fusarium solani* causal agent of root rot disease in olive trees. *Biol. Control* **2017**, *110*, 70-78.

6. Bompadre, M.J.; Pérgola, M.; Fernández Bidondo, L.; Colombo, R.P.; Silvani, V.A.; Pardo, A.G.; Ocampo, J.A.; Godeas, A.M. Evaluation of arbuscular mycorrhizal fungi capacity to alleviate abiotic stress of olive (*Olea europaea* L.) plants at different transplant conditions. *Sci. World J.* **2014**, *6*, 378950.

7. Bompadre, M.J.; Rios De Molina, M.C.; Colombo, R.P.; Fernandez Bidondo, L.; Silvani, V.A.; Pardo, A.G.; Ocampo, J.A.; Godeas, A.M. Differential efficiency of two strains of the arbuscular mycorrhizal fungus *Rhizophagus irregularis* on olive (*Olea europaea*) plants under two water regimes. *Symbiosis* **2013**, *61*, 105-112.

8. Bouaichi, A.; Benkirane, R.; El-Kinany, S.; Habbadi, K.; Lougraimzi, H.; Sadik, S.; Benbouazza, A.; Achbani, E.H. Potential effect of antagonistic bacteria in the management of olive knot disease caused by *Pseudomonas savastanoi pv. savastanoi*. *J. Microbiol. Biotechnol. Food Sci.* **2019**, *8*, 1035-1040.

9. Boutaj, H.; Meddich, A.; Wahbi, S.; Moukhli, A.; El Alaoui-Talibi, Z.; Douira, A.; Filali-Maltouf, A.; El Modafar, C. Effect of arbuscular mycorrhizal fungi on verticillium wilt development of olive trees caused by *Verticillium dahliae*. *Res. J. Biotechnol.* **2019**, *14*, 79-88.

10. Boutaj, H.; Meddich, A.; Wahbi, S.; Moukhli, A.; El Alaoui-Talibi, Z.; Douira, A.; Filali-Maltouf, A.; El Modafar, C. Improvement of growth and development of olive tree by mycorrhizal autochthonous inoculum. *Res. J. Biotechnol.* **2020**, *15*, 76-84.

11. Bouzoumita, A.; Metoui, M.; Jemni, M.; Kabaeir, N.; Belhouchette, K.; Ferchichi, A. The efficacy of various bacterial organisms for biocontrol of fusarium root rot of olive in Tunisia. *Polish J. Environ. Stud.* **2020**, *29*, 11-16.

12. Briccoli Bati, C.; Santilli, E.; Lombardo, L. Effect of arbuscular mycorrhizal fungi on growth and on micronutrient and macronutrient uptake and allocation in olive plantlets growing under high total Mn levels. *Mycorrhiza* **2014**, *25*, 97-108.

13. Cabanás, C.G.L.; Legarda, G.; Ruano-Rosa, D.; Pizarro-Tobías, P.; Valverde-Corredor, A.; Niqui, J.L.; Triviño, J.C.; Roca, A.; Mercado-Blanco, J. Indigenous *Pseudomonas* spp. strains from the olive (*Olea europaea* L.) rhizosphere as effective biocontrol agents against *Verticillium dahliae*: From the host roots to the bacterial genomes. *Front. Microbiol.* **2018**, *9*, 277.

14. Gómez-Lama Cabanás, C.; Ruano-Rosa, D.; Legarda, G.; Pizarro-Tobías, P.; Valverde-Corredor, A.; Triviño, J.C.; Roca, A.; Mercado-Blanco, J. Bacillales members from the olive rhizosphere are effective biological control agents against the defoliating pathotype of *Verticillium dahliae*. *Agric.* **2018**, *8*, 90.

15. Cabanás, C.G.L.; Schilirò, E.; Valverde-Corredor, A.; Mercado-Blanco, J. The biocontrol endophytic bacterium *Pseudomonas fluorescens* PICF7 induces systemic defense responses in aerial tissues upon colonization of olive roots. *Front. Microbiol.* **2014**, *5*, 427.

16. Cabanás, C.G.L.; Sesmero, R.; Valverde-Corredor, A.; Javier López-Escudero, F.; Mercado-Blanco, J. A split-root system to assess biocontrol effectiveness and defense-related genetic responses in above-ground tissues during the tripartite interaction *Verticillium dahliae*-olive-*Pseudomonas fluorescens* PICF7 in roots. *Plant Soil* **2017**, 417, 433-452.

17. Calvo-Polanco, M.; Sánchez-Castro, I.; Cantos, M.; García, J.L.; Azcón, R.; Ruiz-Lozano, J.M.; Beuzón, C.R.; Aroca, R. Effects of different arbuscular mycorrhizal fungal backgrounds and soils on olive plants growth and water relation properties under well-watered and drought conditions. *Plant Cell Environ.* **2016**, 39, 2498-2514.

18. Carrero-Carrón, I.; Trapero-Casas, J.L.; Olivares-García, C.; Monte, E.; Hermosa, R.; Jiménez-Díaz, R.M. *Trichoderma asperellum* is effective for biocontrol of Verticillium wilt in olive caused by the defoliating pathotype of *Verticillium dahliae*. *Crop Prot.* **2016**, *88*, 45-52.

19. Chatzistathis, T.; Orfanoudakis, M.; Alifragis, D.; Therios, I. Colonization of Greek olive cultivars' root system by arbuscular mycorrhiza fungus: Root morphology, growth, and mineral nutrition of olive plants. *Sci. Agric.* **2013**, *70*, 185-194.

20. Cheffi, M.; Bouket, A.C.; Alenezi, F.N.; Luptakova, L.; Belka, M.; Vallat, A.; Rateb, M.E.; Tounsi, S.; Triki, M.A.; Belbahri, L. *Olea europaea* L. root endophyte *Bacillus velezensis* oee1 counteracts oomycete and fungal harmful pathogens and harbours a large repertoire of secreted and volatile metabolites and beneficial functional genes. *Microorganisms* **2019**, *7*, 314.

21. Chenchouni, H.; Mekahlia, M.N.; Beddiar, A. Effect of inoculation with native and commercial arbuscular mycorrhizal fungi on growth and mycorrhizal colonization of olive (*Olea europaea* L.). *Sci. Hortic.* **2020**, *261*, 108969.

22. Chliyeh, M.; Ouazzani Touhami, A.; Filali-Maltouf, A.; El Modafar, C.; Moukhli, A.; Oukabli, A.; Benkirane, R.; Douira, A. Effect of a composite endomycorrhizal inoculum on the growth of olive trees under nurseries conditions in Morocco. *Int. J. Pure Appl. Biosci.* **2014**, *2*, 1–14.

23. Costa, S.M.L.; Melloni, R. Relationship of arbuscular mycorrhizal fungi and rhizobacteria on the growth of olive tree seedlings (*Olea europaea*). *Cienc. Florest.* **2019**, 29, 169-181.

24. Dag, A.; Yermiyahu, U.; Ben-Gal, A.; Zipori, I.; Kapulnik, Y. Nursery and post-transplant field response of olive trees to arbuscular mycorrhizal fungi in an arid region. *Crop Pasture Sci.* **2009**, *60*, 427-433.

25. El-Shazly, M.M.; Ghieth, W.M. Effect of some biofertilizers and humic acid application on olive seedlings growth under irrigation with saline water. *Alexandria Sci. Exch. J.* **2019**, *40*, 263-279.

26. Ferreira, G.M. dos R.; Melloni, R.; da Silva, L.F. de O.; Martins, F.B.; Gonçalves, E.D. Arbuscular mycorrhizal fungi in seedling development of olive (*Olea europaea* L.) in the south of Minas Gerais, Brazil. *Rev. Bras. Cienc. do Solo* **2015**, *39*, 361-366.

27. Fouad, M.O.; Essahibi, A.; Benhiba, L.; Qaddoury, A. Effectiveness of arbuscular mycorrhizal fungi in the protection of olive plants against oxidative stress induced by drought. *Spanish J. Agric. Res.* **2014**, 12, 763-771.

28. Ghanney, N.; Locantore, P. Potential biocontrol effect of the phylloplane bacterium *Bacillus mojavensis* abc-7 on the olive knot disease. *J. Plant Pathol. Microbiol.* **2016**, *7*, 337.

29. Hafez, O.M.; Saleh, M.A.; El-Lethy, S.R. Response of some seedlings olive cultivars to foliar spray of yeast and garlic extracts with or without vascular arbuscular mycorrhizal fungi. *World Appl. Sci. J.* **2013**, 24, 1119-1129.

30. Haggag, F.; Merwad, M.A.; Shahin, M.F.M.; Fouad, A.A. Effect of NPK and bio-fertilizers as soil application on promoting growth of "Toffahi" olive seedlings under greenhouse condition. *Journal of Agricultural Technology* **2015,** 10, 1607-1617.

31. Hibar, K.; Gamaoun, W.; Triki, M.A. Isolation, identification and biological control of the major pathogens causing root rot and wilt diseases of young olive trees in. *J. New Sci.* **2017**, *39*, 2121–2130.

32. Jiménez-Moreno, M.J.; Moreno-Márquez, M. del C.; Moreno-Alías, I.; Rapoport, H.; Fernández-Escobar, R. Interaction between mycorrhization with *Glomus intraradices* and phosphorus in nursery olive plants. *Sci. Hortic.* **2018**, *233*, 249-255.

33. Kapulnik, Y.; Tsror, L.; Zipori, I.; Hazanovsky, M.; Wininger, S.; Dag, A. Effect of AMF application on growth, productivity and susceptibility to Verticillium wilt of olives grown under desert conditions. *Symbiosis* **2010**, *52*, 103-111.

34. Kara, Z.; Arslan, D.; Güler, M.; Güler, Ş. Inoculation of arbuscular mycorrhizal fungi and application of micronized calcite to olive plant: Effects on some biochemical constituents of olive fruit and oil. *Sci. Hortic.* **2015**, *185*, 219-227.

35. Khabou, W.; Hajji, B.; Zouari, M.; Rigane, H.; Abdallah, F.B. Arbuscular mycorrhizal fungi improve growth and mineral uptake of olive tree under gypsum substrate. *Ecol. Eng.* **2014**, *73*, 290-296.

36. Maksoud, M.A.; El-Shamma, M.S.; Saleh, M.A.; Zaied, N.S.; Hafez, O.M. Effect of different compost sorts and biofertilizers on chemlali olive trees grown in calcareous soil. *Middle East J. Sci. Res.* **2012**, *12*, 1046-1049.

37. Maksoud, M.A.; Saleh, M.A.; Fouad, A.A. The beneficial effect of biofertilizers and antioxidants on olive trees under calcareous soil conditions. **2009**, *5*, 350–352.

38. Maldonado-González, M.M.; Prieto, P.; Ramos, C.; Mercado-Blanco, J. From the root to the stem: Interaction between the biocontrol root endophyte *Pseudomonas fluorescens* PICF7 and the pathogen *Pseudomonas savastanoi* NCPPB 3335 in olive knots. *Microb. Biotechnol.* **2013**, *6*, 275-287.

39. Markakis, E.A.; Tjamos, S.E.; Antoniou, P.P.; Paplomatas, E.J.; Tjamos, E.C. Biological control of Verticillium wilt of olive by *Paenibacillus alvei* strain K165. *BioControl* **2016**, *61*, 293-303.

40. M'barki, N.; Chehab, H.; Aissaoui, F.; Dabbaghi, O.; Attia, F.; Mahjoub, Z.; Laamari, S.; Chihaoui, B.; del Giudice, T.; Jemai, A.; et al. Effects of mycorrhizal fungi inoculation and soil amendment with hydrogel on leaf anatomy, growth and physiology performance of olive plantlets under two contrasting water regimes. *Acta Physiol. Plant.* **2018**, *40*, 116.

41. Mechri, B.; Attia, F.; Tekaya, M.; Cheheb, H.; Hammami, M. Colonization of olive trees (*Olea europaea* L.) with the arbuscular mycorrhizal fungus *Glomus* sp. modified the glycolipids biosynthesis and resulted in accumulation of unsaturated fatty acids. *J. Plant Physiol.* **2014**, *171*,1217-1220.

42. Mechri, B.; Tekaya, M.; Cheheb, H.; Attia, F.; Hammami, M. Accumulation of flavonoids and phenolic compounds in olive tree roots in response to mycorrhizal colonization: A possible mechanism for regulation of defense molecules. *J. Plant Physiol.* **2015**, *185*, 40-43.

43. Meddad-Hamza, A.; Beddiar, A.; Gollotte, A.; Lemoine, M.C.; Kuszala, C.; Gianinazzi, S. Arbuscular mycorrhizal fungi improve the growth of olive trees and their resistance to transplantation stress. *African J. Biotechnol.* **2010**, *9*, 1159-1167.

44. Merwad, M.A.; Shahin, M.F.M.; Haggag, L.F. Optimizing growth of "Picual" olive seedlings by using organic and biofertilizers as soil application under greenhouse condition. *Int. J. ChemTech Res.* **2015**, 8, 36-42.

45. Montero-Calasanz, M.C.; Santamaría, C.; Albareda, M.; Daza, A.; Duan, J.; Glick, B.R.; Camacho, M. Alternative rooting induction of semi-hardwood olive cuttings by several auxin-producing bacteria for organic agriculture systems. *Spanish J. Agric. Res.* **2013**, 11, 146-154.

46. Mulero-Aparicio, A.; Varo, A.; Agustí-Brisach, C.; López-Escudero, F.J.; Trapero, A. Biological control of Verticillium wilt of olive in the field. *Crop Prot.* **2020**, *128*, 104993.

47. Nigro, F.; Antelmi, I.; Labarile, R.; Sion, V.; Pentimone, I. Biological control of olive anthracnose. *Acta Hortic*. **2018**, *1199*, 439-444.

48. Otero, M.L.; Roca, M.; Zapata, R.; Ladux, J.L.; Ortiz, J.; Zanelli, M.; Matías, A.C.; Pérez, B.A. Effect of solarization, organic matter, and *Trichoderma* on the severity of Verticillium wilt in olive trees (*Olea europaea* L.) and soil inoculum density. *Acta Hortic.* **2014**, *1057*, 121-126

49. Ouledali, S.; Ennajeh, M.; Ferrandino, A.; Khemira, H.; Schubert, A.; Secchi, F. Influence of arbuscular mycorrhizal fungi inoculation on the control of stomata functioning by abscisic acid (ABA) in drought-stressed olive plants. *South African J. Bot.* **2019**, *121*, 152-158.

50. Ouledali, S.; Ennajeh, M.; Zrig, A.; Gianinazzi, S.; Khemira, H. Estimating the contribution of arbuscular mycorrhizal fungi to drought tolerance of potted olive trees (*Olea europaea*). *Acta Physiol. Plant.* **2018**, *40*, 81.

51. Peyvandi, M.; Farahani, F.; Hosseini Mazinani, M.; Noormohamadi, Z.; Ataii, S.; Asgharzade, A. *Pseudomonas fluorescent* and its ability to promote root formation of olive microshoots. *Int. J. Plant Prod.* **2010,** *4*, 63-66.

52. Porras-Soriano, A.; Soriano-Martín, M.L.; Porras-Piedra, A.; Azcón, R. Arbuscular mycorrhizal fungi increased growth, nutrient uptake and tolerance to salinity in olive trees under nursery conditions. *J. Plant Physiol.* **2009**, *166*, 1350-1359.

53. Prieto, P.; Navarro-Raya, C.; Valverde-Corredor, A.; Amyotte, S.G.; Dobinson, K.F.; Mercado-Blanco, J. Colonization process of olive tissues by *Verticillium dahliae* and its in planta interaction with the biocontrol root endophyte *Pseudomonas fluorescens* PICF7. *Microb. Biotechnol.* **2009**, *2*, 499-511.

54. Prieto, P.; Schilirò, E.; Maldonado-González, M.M.; Valderrama, R.; Barroso-Albarracín, J.B.; Mercado-Blanco, J. Root hairs play a key role in the endophytic colonization of olive roots by *Pseudomonas* spp. with biocontrol activity. *Microb. Ecol.* **2011**, *32*, 435-445.

55. Ruano-Rosa, D.; Prieto, P.; Rincón, A.M.; Gómez-Rodríguez, M.V.; Valderrama, R.; Barroso, J.B.; Mercado-Blanco, J. Fate of *Trichoderma harzianum* in the olive rhizosphere:

time course of the root colonization process and interaction with the fungal pathogen *Verticillium dahliae*. *BioControl* **2016**, *61*, 269-282.

56. Rosa D.D, Villa F., Silva D.F. and Corbari F. (2018) 'Rooting of semihardwood cuttings of olive: indolbutyric acid, calcium and *Azospirillum brasilense', Comunicata Scientiae,* 9(1):34-40.

57. Sanei, S. Suppression of Verticillium wilt of olive by *Pseudomonas fluorescens*. *Am. J. Exp. Agric.* **2011**, *1*, 294-305.

58. Schilirò, E.; Ferrara, M.; Nigro, F.; Mercado-Blanco, J. Genetic responses induced in olive roots upon colonization by the biocontrol endophytic bacterium *Pseudomonas fluorescens* PICF7. *PLoS One* **2012**, *7*, e48646.

59. Seifi, E.; Teymoor, Y.S.; Alizadeh, M.; Fereydooni, H. Olive mycorrhization: Influences of genotype, mycorrhiza, and growing periods. *Sci. Hortic.* **2014**, *180*, 214-219.

60. Semane, F.; Chliyeh, M.; Talbi, Z.; Touati, J.; Selmaoui, K.; Touhami, A.O.; Filali-Maltouf, A.; Modafar, C. El; Moukhli, A.; Benkirane, R.; et al. Effects of a composite endomycorrhizal inoculum on olive cuttings under the greenhouse conditions. *Int. J. Environ. Agric. Biotechnol.* **2017**, *2*, 1070-1083.

61. Shaheen, S.A.; El Taweel, A.A.; Omar, M.N.A. Effect of inoculation by some plant growth promoting rhizobacteria (PGPR) on production of "Manzanillo" olive trees. *Acta Hortic.* **2014**, *1018*, 245-254.

62. Sidhoum, W.; Fortas, Z. Effect of Arbuscular mycorrhizal fungi on growth of semi-woody olive cuttings of the variety " Sigoise " in Algeria. *Am. J. Res. Commun.* **2013**, *1*, 244-257.

63. Silva, T.F.; Melloni, R.; Melloni, E.G.P.; Gonçalves, E.D. Non-symbiotic diazotrophic bacteria and the rooting of olive semi-hardwood cuttings (*Olea europaea* L.). *Cienc. Florest.* **2017**, 27, 61-71.

64. Slama, H. Ben; Triki, M.A.; Bouket, A.C.; Mefteh, F. Ben; Alenezi, F.N.; Luptakova, L.; Cherif-Silini, H.; Vallat, A.; Oszako, T.; Gharsallah, N.; et al. Screening of the high-rhizosphere competent limoniastrum monopetalum' culturable endophyte microbiota allows the recovery of multifaceted and versatile biocontrol agents. *Microorganisms* **2019**, *7*, 249.

65. Tekaya, M.; Mechri, B.; Mbarki, N.; Cheheb, H.; Hammami, M.; Attia, F. Arbuscular mycorrhizal fungus *Rhizophagus irregularis* influences key physiological parameters of olive trees (*Olea europaea* L.) and mineral nutrient profile. *Photosynthetica* **2017**, *55*, 308-316.

66. Varo, A.; Moral, J.; Lozano-Tóvar, M.D.; Trapero, A. Development and validation of an inoculation method to assess the efficacy of biological treatments against Verticillium wilt in olive trees. *BioControl* **2016**, *61*, 283-292.

67. Varo-Suárez, A.; Raya-Ortega, M.C.; Agustí-Brisach, C.; García-Ortiz-Civantos, C.; Fernández-Hernández, A.; Mulero-Aparicio, A.; Trapero, A. Evaluation of organic amendments from agro-industry waste for the control of verticillium wilt of olive. *Plant Pathol.* **2018**, *67*, 860-870.
